# Exploring the pharmacological mechanisms of icaritin against nasopharyngeal carcinoma *via* network pharmacology and experimental validation

**DOI:** 10.3389/fphar.2022.993022

**Published:** 2022-11-18

**Authors:** Minglu Liu, Tong Hu, Wenfeng Gou, Huajie Chang, Yanli Li, Yiliang Li, Daiying Zuo, Wenbin Hou, Shunchang Jiao

**Affiliations:** ^1^ Department of Medical Oncology, The First Medical Centre, Chinese People’s Liberation Army General Hospital, Beijing, China; ^2^ Tianjin Key Laboratory of Radiation Medicine and Molecular Nuclear Medicine, Institute of Radiation Medicine, Peking Union Medical College and Chinese Academy of Medical Sciences, Tianjin, China; ^3^ Department of Pharmacology, Shenyang Pharmaceutical University, Shenyang, China

**Keywords:** icaritin, nasopharyngeal carcinoma, ROS, senescence, network pharmacology

## Abstract

**Background:** Icaritin is a natural product with a wide range of anti-tumor effects. However, its anti-tumor mechanism has not been thoroughly studied. This study examined the inhibitory effect of icaritin on nasopharyngeal cancer and its underlying mechanism using network pharmacology along with *in vivo* and *in vitro* experiments.

**Methods:** MTT and clone formation assays were used to detect the effects of icaritin on the viability and proliferation of nasopharyngeal carcinoma cells, followed by the construction of a HONE1 xenograft tumor model to evaluate the anti-tumor efficacy of icaritin *in vivo*. A public database was used to predict prospective targets, built a protein-protein interaction (PPI) network, and analyze gene enrichment and biological processes. Based on network pharmacological data, cell cycle-related proteins were identified using western blotting. Besides, cell cycle distribution, apoptosis, and intracellular reactive oxygen species (ROS) generation were identified using flow cytometry. In addition, SA-β-Gal staining was performed to detect cellular senescence, and western blotting was performed to detect the expression of P53, P21, and other proteins to verify key signaling pathways.

**Results:** Icaritin effectively inhibited the viability and proliferation of nasopharyngeal carcinoma cell lines and showed good anti-tumor activity against HONE1 nasopharyngeal carcinoma cells *in vivo*. Key protein targets, including AKT1, HSP90AA1, CDK4, CCND1, and EGFR, were screened using PPI network topology analysis. GO and KEGG analysis revealed that the cell cycle, p53 signaling, and cell senescence pathways may be the main regulatory pathways. Flow cytometry and western blot experiments showed that icaritin caused S-phase arrest and promoted an increase in ROS. SA-β-Gal staining showed that icaritin significantly induced cellular senescence, and western blotting showed that the expression of senescence-related proteins p53 and P21 increased significantly. Moreover, inhibition of ROS levels by N-Acetylcysteine (NAC) enhanced cell viability, reversed cellular senescence and reduced cellular senescence-associated protein expression.

**Conclusion:** The results of network pharmacological analysis and *in vivo* and *in vitro* experiments showed that icaritin effectively inhibited the growth of nasopharyngeal carcinoma cells, promoted ROS production, induced cellular senescence, and inhibited tumor cells, which are related to the regulation of P53/P21 signal pathway.

## Introduction

A common malignant tumor in otolaryngology, nasopharyngeal carcinoma develops from the nasopharynx’s epithelial cells (Guo et al., 2019). Its incidence is influenced by several factors and has a distinct geographical distribution, mainly in East and Southeast Asia and southern China, and the condition is currently treated with surgery and radiation therapy (Guo et al., 2019; Perri et al., 2019). Although surgery and radiation therapy have slowed down the progression of tumors in some patients, 30%–60% of patients still develop local recurrence and/or distant metastases (Perri et al., 2019); therefore, finding treatments to enhance the sensitivity of nasopharyngeal carcinoma and improve its prognosis is urgently needed.

Chinese herbal medicines have been widely used by the medical community because of their naturally and low-toxicity characteristics (Yang et al., 2021). Epimedium sagittatum is a herb of *Epimedium sagittatum* (Sieb. et Zucc.) Maxim. or *Epimedium brevicornum* Maxim. of the family Berberidaceae, which tonifies the liver and kidney, reinforces the muscles and bones, and dispel wind and dampness (Kang et al., 2015). One of its primary active ingredients, icaritan, has a variety of pharmacological actions, including anti-inflammatory, anti-osteoporosis, and anti-depressive activity. (Xue et al., 2016; Ma et al., 2022; Sun et al., 2022).

On 10 January 2022, the National Medical Products Administration approved the marketing of icaritin through a priority review for the treatment of patients with advanced unresectable hepatocellular carcinoma not receiving systemic therapy, indicating that icaritin has good anti-tumor effects. In recent years, icaritin has been increasingly used in oncology. According to the literature, icaritin has been reported to play a pivotal role in a number of malignancies such as liver cancer (Sun et al., 2015; Yu et al., 2020), breast cancer (Guo et al., 2011; Wang et al., 2017; Yin et al., 2020), oral squamous cell carcinoma (Jin et al., 2017; Yang et al., 2017), cervical cancer (Chen et al., 2019), glioblastoma (Liu et al., 2018; Li et al., 2020), and colon cancer (Li et al., 2016), but nasopharyngeal cancer has not been mentioned.

Investigating the mechanism of action and impact of icaritin on nasopharyngeal cancer is the goal of this study, we performed *in vitro* cell viability assays and constructed an *in vivo* xenograft tumor model and then analyzed and predicted the potential targets and pathways of icaritin in the treatment of nasopharyngeal carcinoma using network pharmacology technology. Finally, we validated the potential targets and pathways of icaritin using flow cytometry and western blotting to reveal the mechanism of action of icaritin in the treatment of nasopharyngeal carcinoma and provide theoretical support for the clinical treatment of nasopharyngeal carcinoma with icaritin.

## Materials and method

### Reagents

Icaritin was purchased from Sigma-Aldrich (MO, United States). Fetal bovine serum (FBS) was purchased from Gibco (Waltham, MA). 3-(4, 5-dimethylthiazol-2-yl)-2, 5-diphenyltetrazolium bromide (MTT), crystal violet, a cell-based ROS assay kit, N-acetylcysteine (NAC), and a senescence-associated β-galactosidase (SA-β-Gal) kit were obtained from Beyotime Biotechnology (Beijing, China). Annexin V-FITC/PI and propidium iodide (PI) were purchased from KeyGEN BioTECH (Nanjing, China). The primary antibody against γ-H2AX was obtained from Cell Signaling Technology (MA, United States). Primary antibodies against ki67, GAPDH, PARP1, cyclin D1, CDK2, CDK4, Bax, Bcl-2, P53, P21, p-EGFR, EGFR, p-AKT, AKT, and HIF1α were obtained from Proteintech (Rockville, MD, United States). Antibody against horseradish peroxidase (HRP)-conjugated secondary antibodies (goat-anti-mouse or goat-anti-rabbit) were obtained from ZSGB-BIO (Beijing, China).

### Cell culture

The human nasopharyngeal carcinoma cell lines HONE1 and HNE1 were purchased from Zhongqiaoxinzhou Biotech (Shanghai, China). HONE1 and HNE1 cells were cultured in DMEM and RPMI-1640 media containing 1% penicillin-streptomycin and 10% FBS, respectively. Both cell lines were cultured at 37°C in a 5% CO_2_ atmosphere.

### MTT assay

HONE1 and HNE1 cells were seeded into 96 well plates at the density of 3,000 cells per well, and different concentrations of icaritin were added to the cells after 24 h culture. After 72 h, 20 μl MTT solution (5.0 mg/ml) was added and incubated at 37°C for 4 h, then added 150 μl of DMSO. The optical density was measured at 570 nm using a microplate reader (Tecan, Switzerland).

### Colony formation assay

HONE1 and HNE1 cells were seeded into 6 well plates, 5,000 cells per well. After 24 h incubation, the cells were treated with icaritin (10, 20, or 40 μM) for 48 h. The fresh culture medium was replaced and cultured for 2 weeks and stained with 0.1% crystal violet solution for 10 min. The colonies were photographed using a digital camera (SONY, JPN).

### Target prediction of icaritin and nasopharyngeal carcinoma

SMILES of icaritin were obtained by searching Pubchem (https://pubchem.ncbi.nlm.nih.gov/) and imported into SwissTargetPrediction (http://www.swisstargetprediction.ch/), species selection of *Homo sapiens*, and prediction of icaritin targets. The HERB database was searched for icaritin targets and duplicate targets were merged and removed.

The DISGENET (https://www.disgenet.org/) and TTD databases (http://db.idrblab.net/ttd/) were searched with “nasopharyngeal carcinoma” as the keyword. Then, the targets were merged and duplicates removed to obtain candidate targets for nasopharyngeal carcinoma.

### Construction and analysis of target network

The icaritin and nasopharyngeal carcinoma targets were imported into UniProtKB (http://www.uniprot.org/) to unify the target names. The targets were imported into VENNY2.1 (https://bioinfogp.cnb.csic.es/tools/venny/) to obtain the intersection targets of the compound and disease. The intersecting targets were imported into STRING (https://cn.string-db.org/cgi/input.pl), “*Homo sapiens*” was selected as the species, and medium confidence >0.4 was selected as the minimum interaction threshold, unlinked targets were hidden, and other parameters were kept at default settings. The tsv file was saved after updating and imported into Cytoscape 3.7.1. Then, network analysis was performed, and the clusters with high correlation were calculated using the MCODE plugin.

### GO and KEGG pathway enrichment analysis

Icaritin-nasopharyngeal carcinoma intersection targets were imported into the Metascape database (https://metascape.org/), and the species was set as “*Homo Sapiens*”. The three modules of GO: molecular function, biological process, and cell composition were selected for GO enrichment analysis, and KEGG was selected for pathway analysis. The KEGG pathways of *p* < 0.01 were considered significant, and the results of enrichment analysis were visualized using the microbiology online mapping platform (http://www.bioinformatics.com.cn/).

### Cell cycle analysis

HONE1 and HNE1 cells were seeded into 60 mm cell culture dishes at a density of 3 × 10^5^ cells. After 24 h incubation, the cells were treated with 10, 20, or 40 μM icaritin for 24 h. The cells were then collected and fixed with pre-cooled 70% ethanol for 48 h, then stained with PI working solution at room temperature for 30 min. The cell cycle distribution of each group was analyzed using a FACScan flow cytometer (Becton-Dickinson, NJ, United States).

### Apoptosis assay

HONE1 and HNE1 cells were seeded into 60 mm cell culture dishes at a density of 3 × 10^5^ cells. After 24 h incubation, the cells were treated with 10, 20, or 40 μM icaritin for 48 h. The cells were then collected and stained with Annexin V-FITC/PI working solution for 15 min in the dark. The apoptotic cell ratio was determined using a FACScan flow cytometer.

### Western blot

HONE1 and HNE1 cells were collected after treatment with different concentrations of icaritin for 24 h or 48 h or preated with NAC (5 mM) for 1 h before administration. After lysis, the supernatant was collected and the concentration was quantified by BCA Protein Assay Kit. After SDS-PAGE protein electrophoresis, transferring to PVDF membranes, and blocking with 5% non-fat milk, the membrane was incubated with primary antibodies at 4°C for 12 h, and then incubated with the corresponding secondary antibody at room temperature for 2 h. The protein bands were detected using an ECL kit, and the band density was analyzed by Scion Image software.

### Reactive oxygen species measurement

After treatment with icaritin (10, 20, and 40 μM) for 24 h, the cells were incubated with 10 μM dichlorofluorescin diacetate (DCFH-DA) at 37°C for 20 min in a 5% CO_2_ atmosphere. Then the cells were collected and analyzed using FACScan flow cytometer. The average DCF fluorescence intensity was used to express the intracellular ROS level in each group.

### SA-β-gal analysis

HONE1 and HNE1 cells were seeded in 6 well plates, 5 × 10^4^ cells per well. After 24 h incubation, the cells were treated with 10, 20, or 40 μM icaritin for 24 h. The cells were then stained with SA-β-Gal solution overnight. Six separate fields were counted under a microscope in each group.

### Nude mice xenograft model

BALB/c Nude mice (6-weeks-old) were purchased from HFK Bioscience Co., Ltd. (Beijing, China). The Institutional Animal Care and Use Committee at the Institute of Radiation Medicine approved all animal experiments in this study. HONE1 cells (5 × 10^6^) were injected subcutaneously into the left flanks of the mice. Tumor volume (mm^3^) = 1/2 × length × width^2^. When the tumor volume reached 100 mm^3^, the mice were administered 50 or 100 mg/kg of oral icaritin daily. Tumor volume and body weight were recorded every 2 days. After 14 days, the tumor tissues were harvested and fixed in formalin for immunohistochemistry.

### Immunohistochemistry

The tissue sections were dewaxed with xylene and hydrated, then incubated with 3% H_2_O_2_ at room temperature for 20 min, and 5% bovine serum albumin for blocking at room temperature for 30 min. The primary antibody against Ki67 was added and incubated overnight at 4°C, and then the secondary antibody was added and incubated at 37°C for 1 h. Then stained with DAB and hematoxylin. Finally, the sections were dehydrated, transparent, and sealed with neutral gum.

### Statistical analysis

Statistical analyses were performed using SPSS 23.0. The data are expressed as mean ± standard deviation (SD). Statistical comparisons were performed using the Student’s t-test and one-way ANOVA. *p* < 0.05 was considered to be statistically significant.

## Results

### Icaritin inhibits the proliferation of nasopharyngeal carcinoma cells

**FIGURE 1:**
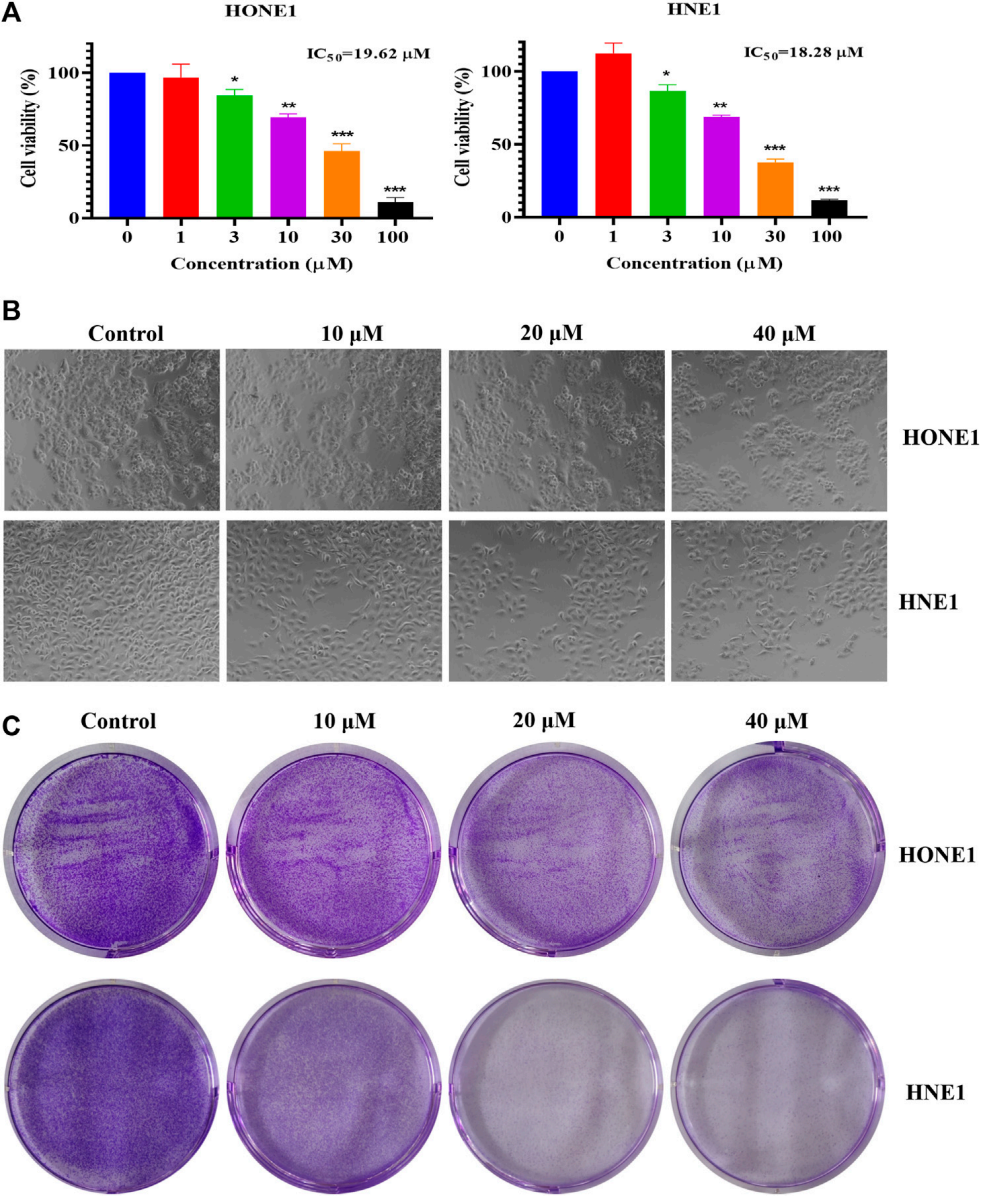
Effects of icaritin on HONE1 and HNE1 cell proliferation. **(A)** Cell viability of HONE1 and HNE1 was determined by MTT assay after treatment with icaritin (0–100 μM) for 72 h. **(B)** Cellular morphology of HONE1 and HNE1 cells after treatment with icaritin (10, 20, or 40 μM) for 48 h. **(C)** Effect of icaritin (10, 20, or 40 μM) on colony formation after 48 h and continuous cultured for 2 weeks. ^*^ *p* < 0.05, ^**^ *p* < 0.01, ^***^ *p* < 0.001 *versus* control.

To explore the effect of icaritin on nasopharyngeal carcinoma, two nasopharyngeal carcinoma cell lines, HONE1 and HNE1 were chosen as the study’s two nasopharyngeal cancer cell lines. According to MTT data, icaritin significantly and dose-dependently reduced the viability of HONE1 and HNE1 cells (Figure 1A). The cell morphology showed that the cell density decreased gradually with the increase of patchouli concentration, and the cell morphology did not change significantly (Figure 1B). A clone formation assay was carried out to further confirm the impact of icaritin on cell proliferation. The results revealed that icaritin suppressed cell growth in a dose-dependent way (Figure 1C). In addition, we explored whether icaritin induced apoptosis in nasopharyngeal carcinoma cells, and the results showed that icaritin induced only slight apoptosis, suggesting that apoptosis may not be the main anti-tumor mechanism of icaritin (Supplementary Figure S1). The results suggest that icaritin may exert anti-tumor effects by inhibiting the proliferation of nasopharyngeal carcinoma cells.

### Icaritin inhibits the tumor growth of nasopharyngeal carcinoma *in vivo*

**FIGURE 2:**
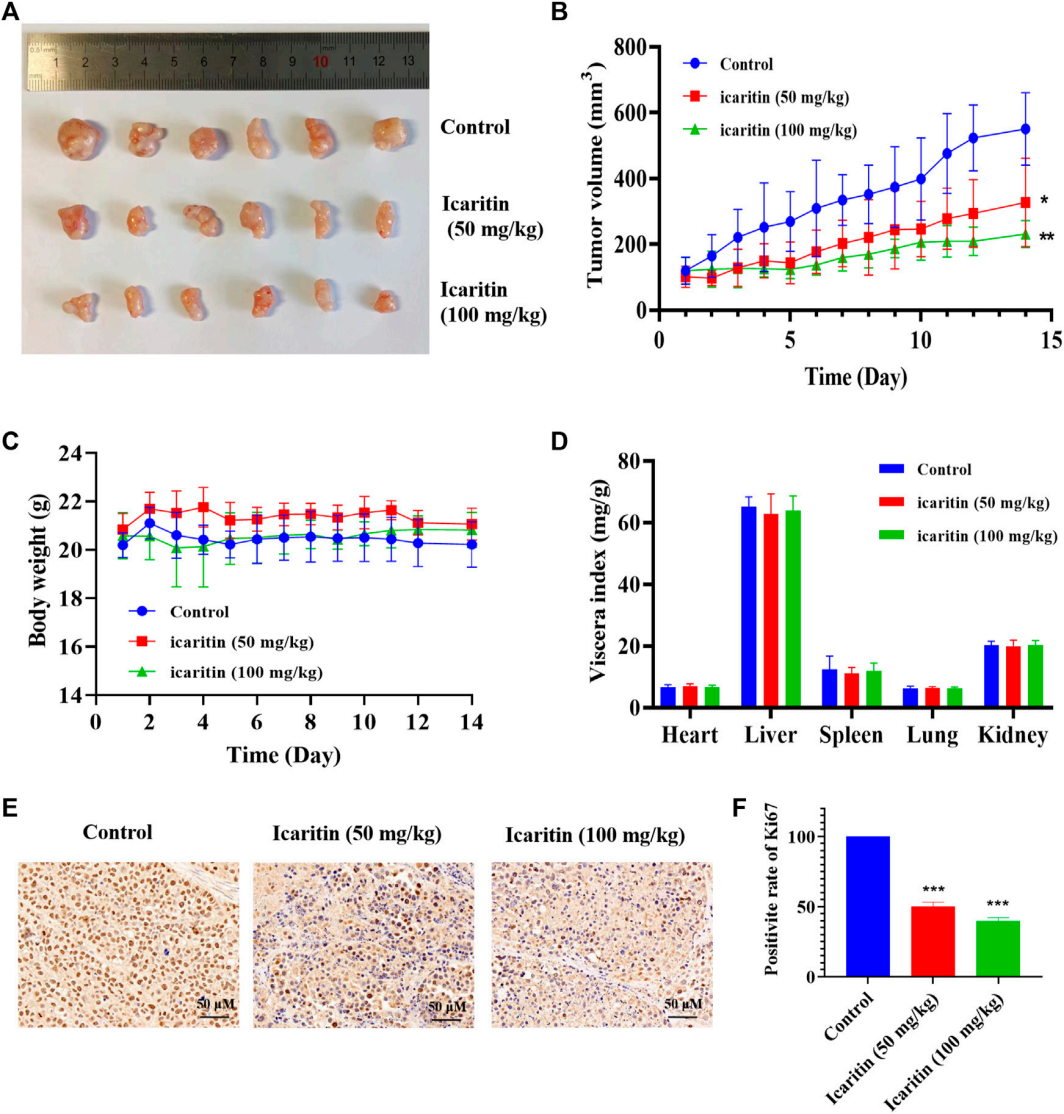
Icaritin elicits a potent antitumor effect *in vivo*. **(A)** Images of resected xenograft tumor samples of HONE1-injected nude mouse. **(B)** Tumor growth curve of mice in each group, *n* = 6. **(C)** Body weight curve of mice in each group, *n* = 6. **(D)** The viscera index of the main organs of mice in each group. **(E)** Representative photomicrographs of ki67 staining in the control and icaritin (50, 100 mg/kg) groups. Scale bars, 50 μm. **(F)** The percentage of Ki67 positive cells.^*^ *p* < 0.05, ^**^ *p* < 0.01, ^***^ *p* < 0.001 *versus* control.

We conducted a xenograft tumor experiment to further explore the anti-tumor effect of icaritin *in vivo*. The results indicated that the tumor volumes in the low (50 mg/kg) and high (100 mg/kg) dose icaritin treatment groups were significantly smaller than those in the blank group (Figures 2A,B), and the tumor growth rate was significantly slower (Figure 2B). In addition, our analysis revealed no significant changes in body weight in the icaritin-treated group compared with that in the blank group (Figure 2C), and organ index analysis showed no significant differences in the heart, liver, spleen, lung, and kidney indices in the icaritin-treated group compared with the corresponding values in the control group (Figure 2D). We then detected the proliferation marker protein ki67 by immunohistochemistry (Liu et al., 2021), and the results showed that the expression of ki67 was significantly inhibited in the icaritin-treated group, which was consistent with the results of *in vitro* experiments, further demonstrating that icaritin can inhibit the proliferation of nasopharyngeal carcinoma (Figures 2E,F). These results suggested that icaritin has good *in vivo* anti-tumor effects and low toxicity.

### Icaritin-nasopharyngeal carcinoma protein-protein interaction network

**FIGURE 3:**
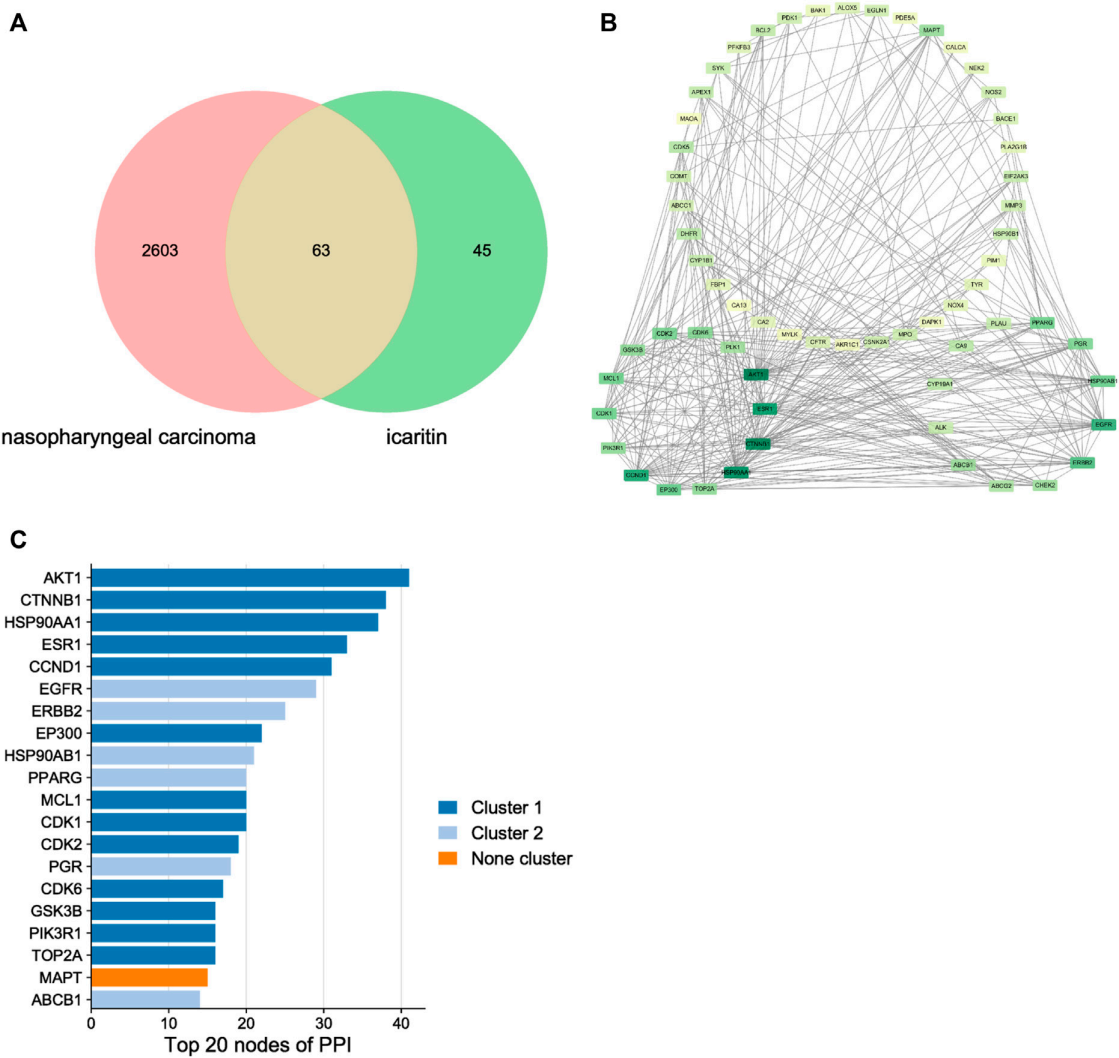
The network pharmacology of icaritin in the treatment of nasopharyngeal carcinoma (NPC). **(A)** Venn diagram of icaritin-NPC. **(B)** Protein-protein interaction network and hub genes of icaritin in the treatment of NPC. **(C)** Top 20 target genes of icaritin in the treatment of NPC.

Through target prediction, database search, and literature research, a total of 108 potential action targets of icaritin and 2,666 nasopharyngeal cancer-related targets were obtained, and after disease-component intersection analysis, a total of 63 intersection targets that may be related to icaritin treatment of nasopharyngeal cancer were identified (Figure 3A; Supplementary Table S1). To identify the core targets of icaritin for the treatment of nasopharyngeal cancer, we used STRING to construct the intersection targets of the PPI network, and the results were analyzed using Cytoscape. The PPI network is shown in Figure 3B, in which the rectangular nodes represent the target proteins and the connecting lines between the nodes represent the existence of interactions between the two proteins. The results showed that the number of interconnected nodes was 62, the number of edges was 365, and average node degree was 11.6. The top 20 interacting hub proteins were obtained based on sorting of the fractional values (Figure 3C). Two core networks were generated by hub gene screening using the MCODE plugin of Cytoscape, and AKT1, CTNNB1, HSP90AA1, ESR1, CCND1, and EGFR were identified as key hub proteins, which may play an important role in the efficacy of icaritin in the treatment of nasopharyngeal carcinoma.

### GO and KEGG pathway enrichment analysis

**FIGURE 4:**
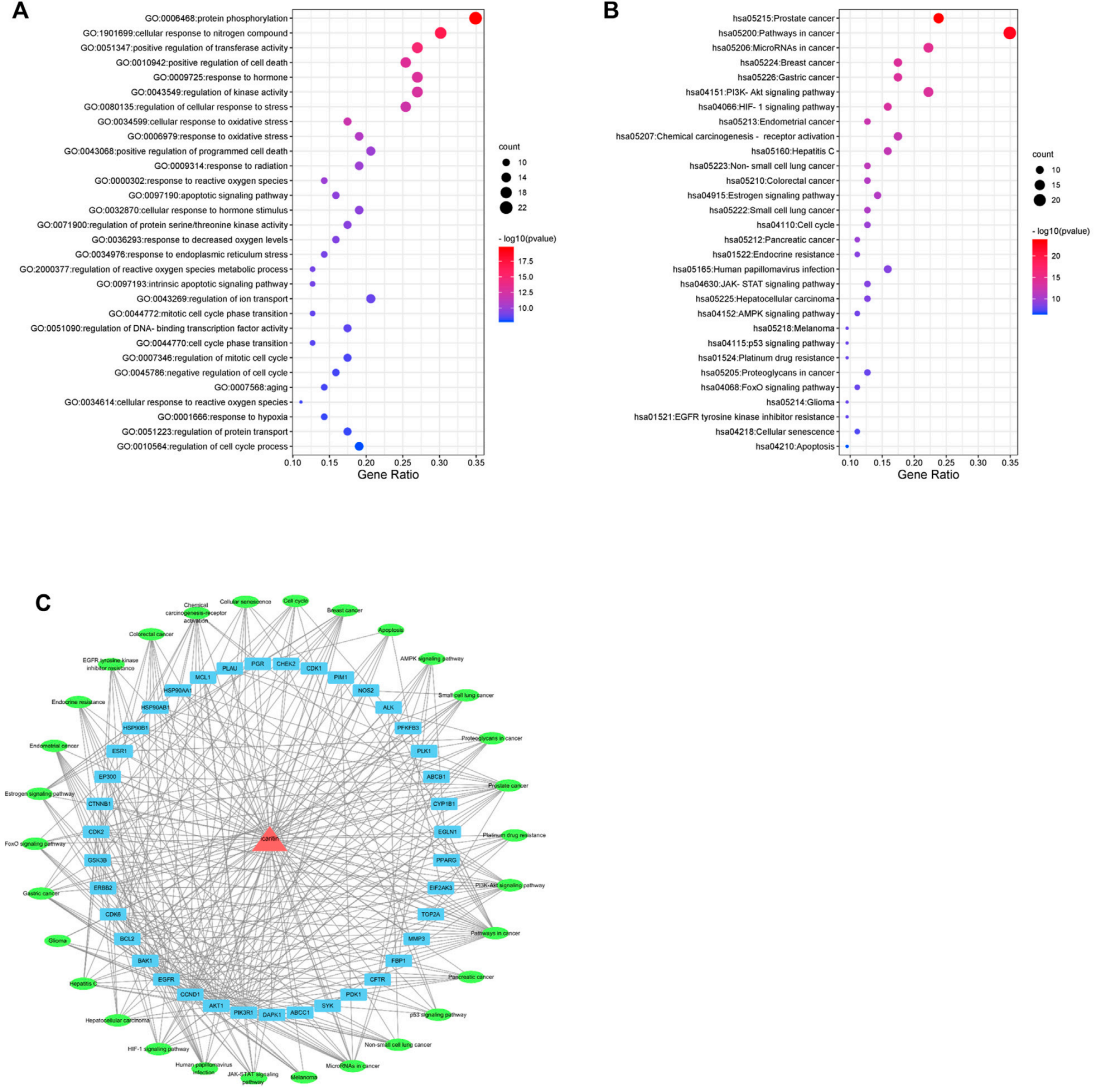
Network pharmacology analysis of 30 validated constituents of icaritin. **(A)** GO enrichment of the key genes in response to icaritin treatment in NPC. **(B)** KEGG enrichment of potential targets of icaritin in the treatment of NPC. **(C)** Icaritin-target-pathway network in the anti-tumor effects of icaritin.

The top 30 enriched GO biological processes (GOBP) and KEGG pathway were selected in ascending order of the *p*-value for visualization (Figures 4A,B). The results showed that GOBPs were mainly related to protein phosphorylation, transferase activity regulation, kinase activity regulation, oxidative stress response, apoptosis, reactive oxygen species metabolism, cell cycle phase transition and cellular senescence (Figure 4A). The pathways related to the anti-nasopharyngeal carcinoma effect of icaritin mainly involved prostate cancer, breast cancer, and other cancer signaling pathways, PI3K-Akt signaling pathway, HIF-1 signaling pathway, estrogen signaling pathway, cell cycle, p53 signaling pathway, cell senescence, and apoptosis (Figure 4B). Combining the results of PPI network analysis of intersecting targets, MCODE core target screening, and KEGG enrichment analysis, we constructed an “icaritin-intersecting target-pathway” network (Figure 4C).

### Icaritin induces S-phase arrest in HONE1 and HNE1 cells

**FIGURE 5:**
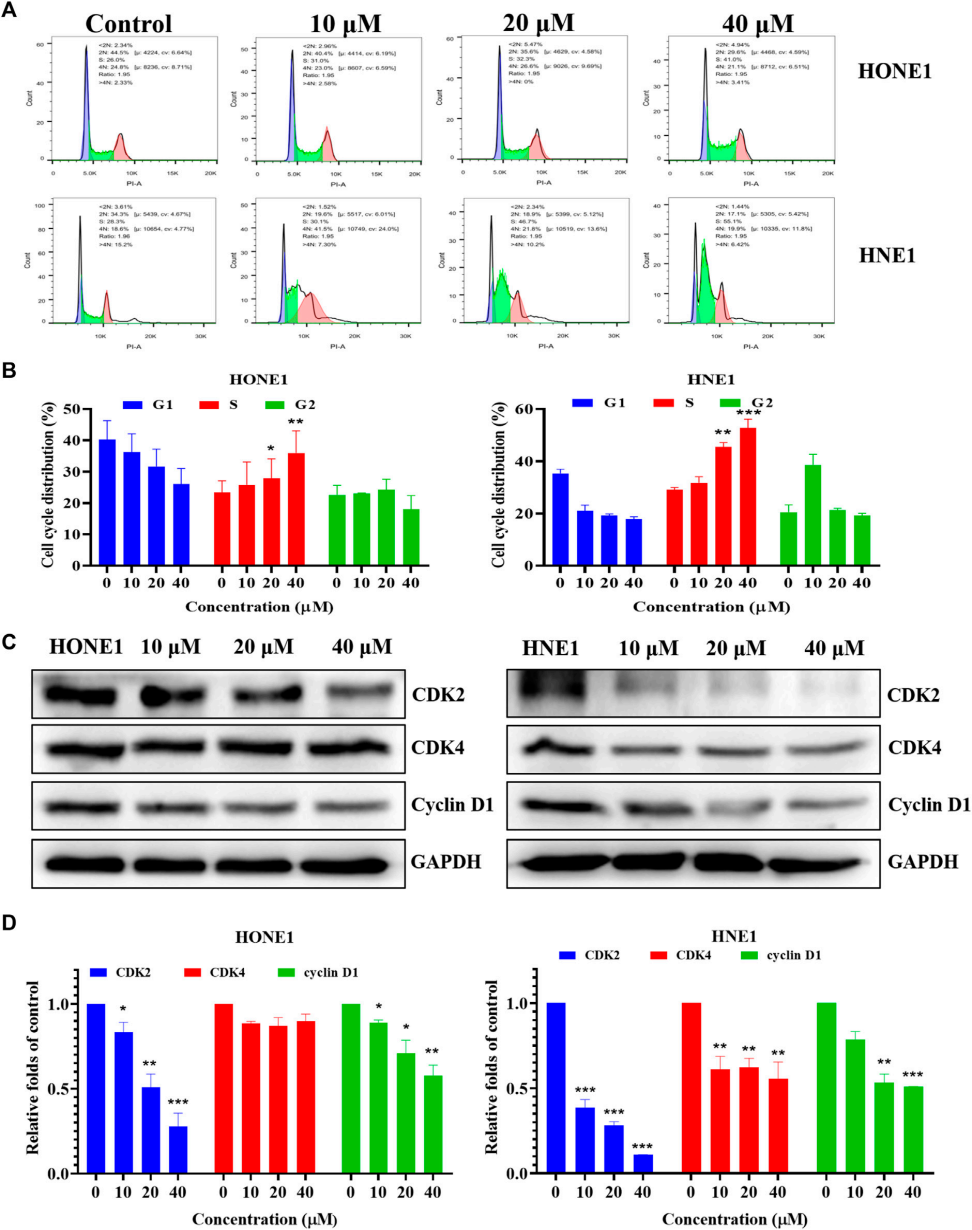
Icaritin arrests HONE1 and HNE1 cell cycles. **(A)** The cell cycle distribution of HONE1 and HNE1 cells was measured by flow cytometry after treatment with icaritin (10, 20, or 40 μM) for 24 h. **(B)** The percentage of cell cycle distribution. **(C)** The expression of G1/S phase-related protein in HONE1 and HNE1 cells was measured by western blotting after treatment with icaritin (10, 20, or 40 μM) for 24 h. **(D)** The quantitative analysis of protein bands. *p < 0.05, **p < 0.01, ***p < 0.001 *versus* control.

Based on the results of the network pharmacology analysis, we examined the changes in PI3K/AKT, EGFR and HIF-1 signaling pathways, and the results showed that these pathway-related proteins were not significantly altered, and we speculate that icaritin may exert anti-tumor effects through other pathways (Supplementary Figure S2). The clone formation results showed that icaritin could significantly inhibit cell proliferation, and we speculate that it is involved in regulating the cell cycle (Figure 1C). We examined the impact of icaritin on the cell cycle of HONE1 and HNE1 cells by flow cytometric analysis in order to further understand the growth inhibition mechanism of icaritin on nasopharyngeal cancer cells. As shown in Figures 5A,B, cells in the S-phase increased significantly after treatment with icaritin, with the proportion of S-phase HONE1 cells increasing from 26% to 41% and the proportion of S-phase HNE1 cells increasing from 28.3% to 55.1%, indicating that icaritin arrested the cells in the S-phase. In order to further clarify the mechanism underlying the S-phase arrest put on by icaritin, we looked at the expression of related proteins in combination with important regulatory targets like CDK4 that were discovered using network pharmacology research. The results showed that icaritin downregulated the expression of cyclin D1, CDK2, and CDK4 in a dose-dependent manner (Figures 5C,D), further demonstrating that icaritin caused S-phase arrest in nasopharyngeal carcinoma cells.

### Icaritin induces HONE1 and HNE1 cell senescence

**FIGURE 6:**
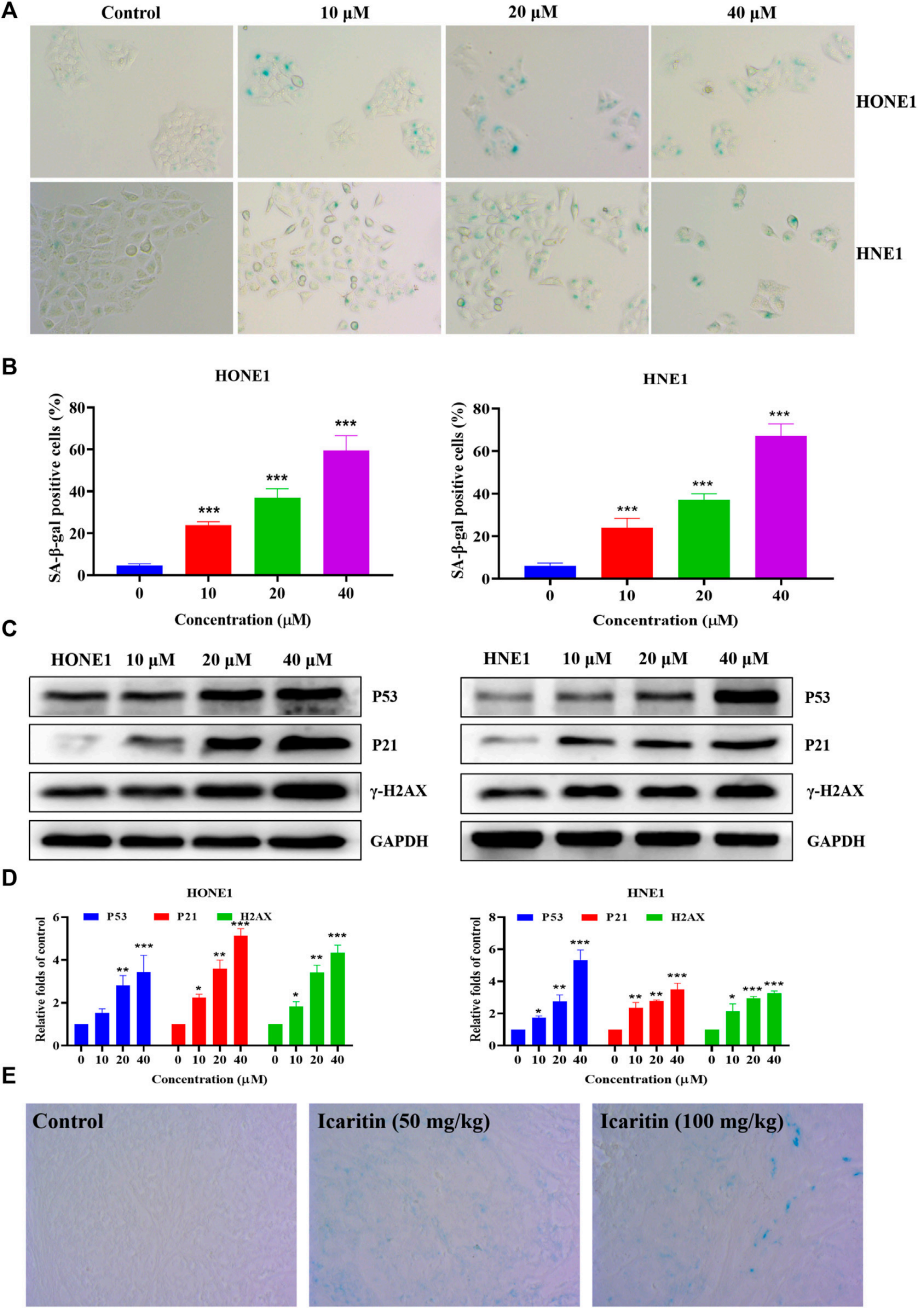
Icaritin induces senescence in the HONE1 and HNE1 cells. **(A)** Representative photomicrographs of SA-β-Gal staining in the control cells and icaritin-treated cells (10, 20, or 40 μM) for 48 h. Scale bars, 10 μm. **(B)** The percentage of SA-β-Gal positive cells. **(C)** The expression of the senescence-related protein in HONE1 and HNE1 cells was detected by western blotting after treatment with icaritin (10, 20, or 40 μM) for 48 h. **(D)** The quantitative analysis of protein bands. **(E)** Representative photomicrographs of SA-β-Gal staining in the control and icaritin (50, 100 mg/kg) groups. Scale bars, 10 μm*p < 0.05, **p < 0.01, ***p < 0.001 *versus* control.

Previous flow cytometry results have shown that icaritin causes cellular S-phase arrest. The cell cycle regulates cell differentiation and proliferation and is also closely related to cellular senescence (Shi et al., 2021; Tubita et al., 2022). The induction of cellular senescence is one of the mechanisms of anti-tumor drugs. We further explored whether icaritin induces senescence in nasopharyngeal carcinoma cells. β-galactosidase is a marker of cellular senescence (Cai et al., 2020; Yu et al., 2022). We treated HONE1 and HNE1 cells with different concentrations of icaritin for 48 h and performed SA-β-Gal staining, which revealed a significant increase in cellular senescence after the treatment with icaritin (Figures 6A,B). To further demonstrate the mechanism underlying cell senescence induction by icaritin, the key proteins P53 and P21, which regulate cell senescence, were detected by western blot in combination with the analysis of the previous network pharmacological data. The results revealed that the expression levels of P53 and P21 proteins gradually increased with an increase in icaritin concentration (Figures 6C,D). In addition, *in vivo* SA-β-Gal staining results showed that senescent cells were significantly increased in the tumor tissues of the iacritin-treated group, and a significant increase in the expression of senescence-related proteins such as P53 in the tumor tissues, consistent with the *in vitro* cellular results (Figure 6E; Supplementary Figure S3), further demonstrating that icaritin exerts its anti-tumor effects by inducing nasopharyngeal carcinoma cell senescence.

### Icaritin increases the reactive oxygen species level of HONE1, and HNE1 cells

**FIGURE 7:**
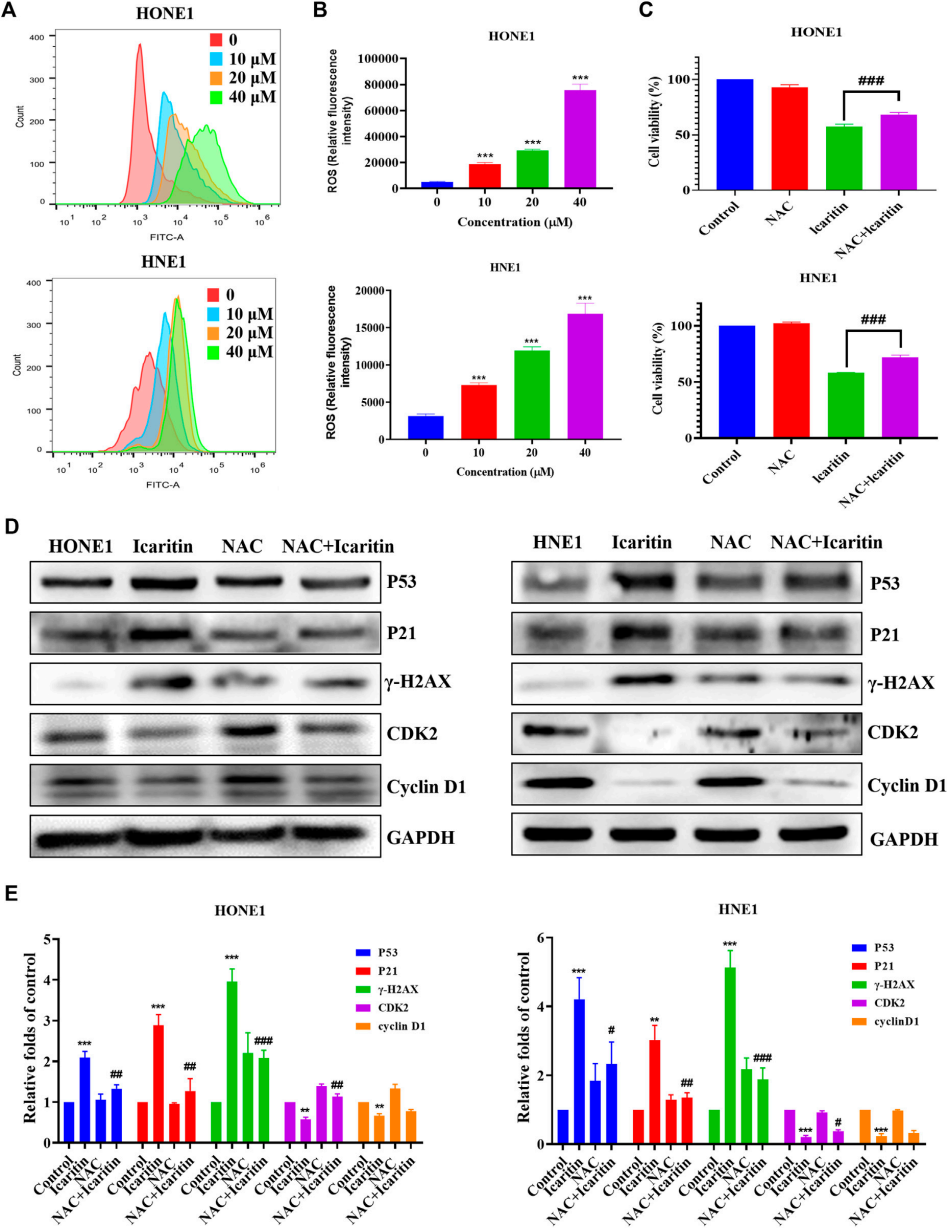
Effect of icaritin on the generation of ROS. **(A)** The ROS levels in icaritin-treated HONE1 and HNE1 cells were assessed using DCF biomarkers and analyzed using flow cytometry. **(B)** Statistical analysis of ROS level in each group. **(C)** Cell viability after treatment with icaritin (20 μM), NAC (5 mM), or a combination of icaritin and NAC for 24 h and determined using the MTT assay. **(D)** The expression of the cell cycle and senescence-related protein in HONE1 and HNE1 cells was detected by western blotting after treatment with icaritin (20 μM), NAC (5 mM) or a combination of icaritin with NAC for 24 h. **(E)** The quantitative analysis of protein bands. **p < 0.01, ***p < 0.001 *versus* control; ^#^ *p* < 0.05, ^##^ *p* < 0.01, ^###^ *p* < 0.001 *versus* icaritin.

Several studies have shown that icaritin increases ROS production in tumor cells (Wu et al., 2014; Wang et al., 2019). To investigate whether icaritin induces an increase in ROS in nasopharyngeal carcinoma cells, we performed cell staining using a DCFH-DA probe and measured the ROS fluorescence intensity by flow cytometry. The results showed that icaritin significantly promoted ROS production in a dose-dependent manner (Figures 7A,B). To further validate the role of ROS in the anti-tumor effects of icaritin, we selected the ROS inhibitor NAC for reverse validation, and the MTT results showed that cell viability was significantly increased in the NAC and icaritin combination group compared with that in the icaritin alone group (Figure 7C). Western blot results showed that inhibition of ROS production decreased the expression of senescence-associated proteins P53 and P21 and restored the expression of CDK2 (Figures 7D,E). In addition, flow cytometry and SA-β-gal staining results showed that inhibition of ROS ameliorated icaritin-induced cellular S-phase arrest and cellular senescence (Supplementary Figure S4). These results suggest that icaritin exerts its anti-tumor effects by increasing ROS levels.

## Discussion

Icaritin, an extract of the traditional Chinese medicine Epimedium sagittatum, belongs to the class of flavonoids (Mo et al., 2021) and has anti-tumor effects in a variety of tumors. However, there is a lack of research on its effect on nasopharyngeal carcinoma. In this study, the anti-tumor effect of icaritin on nasopharyngeal carcinoma was investigated *in vivo* and *in vitro*. The results showed that icaritin had good anti-tumor effects in both *ex vivo* and *in vivo* evaluations. The apoptotic results showed that icaritin induced slight apoptosis in nasopharyngeal carcinoma cells. We thus speculated that the induction of apoptosis may not be the main anti-tumor mechanism of icaritin. Network pharmacology integrates classical pharmacology, molecular biology, bioinformatics, and computer technology to provide ideas for the study of compound targets, mechanisms of action, and drug-target interactions (Yang et al., 2021a; Wang et al., 2022). In this study, we used network pharmacology to predict the possible anti-tumor targets and mechanism of action of icaritin and validated them using MTT, flow cytometry, and western blotting on the basis of data mining analysis and virtual prediction. This laid the foundation for the further development of icaritin.

The PPI network analysis showed that the hub targets of icaritin were mainly cell cycle-related proteins (CCND1, CDK1, CDK2, and CDK6), molecular chaperones (HSP90AA1 and HSP90AB1), tyrosine kinase family members (EGFR and ERBB2), P53/P21 and PI3K/AKT. Based on the results of the network pharmacological analysis, we examined the expression of p-AKT and p-EGFR, and it turned out that these proteins were not significantly altered, and we hypothesized that icaritin exerted its antitumor effects through other pathways. *In vitro* cellular assays revealed that icaritin inhibited the proliferation of nasopharyngeal carcinoma cells, and combined with the results of network pharmacology data, icaritin had a regulatory effect on the cell cycle of nasopharyngeal carcinoma cells. Using flow cytometry and western blotting, we found that icaritin induced cellular S-phase arrest, which is the phase of DNA synthesis, indicating that it blocked DNA synthesis was blocked. To further detect DNA damage, we examined γ-H2AX, a marker protein of DNA damage, and the results showed that the expression of γ-H2AX protein gradually increased with increasing patchouli concentration. These results suggest that icaritin causes DNA damage in nasopharyngeal carcinoma cells, induces cellular S-phase arrest, and inhibits cell proliferation to exert anti-tumor effects.

Cellular senescence, induced when cells undergo irreversible cycle arrest, is one of the main ways to treat tumor (Baek et al., 2020; Rodenak-Kladniew et al., 2020; Liu et al., 2022). Network pharmacological analysis revealed that the cellular senescence pathway is closely related to the anti-tumor effect of icaritin; therefore, we further explored whether icaritin induced cellular senescence in nasopharyngeal carcinoma cells. SA-β-Gal staining *in vitro* and *in vivo* showed that icaritin significantly induced cellular senescence. We then examined the key signaling pathway of senescence regulation, P53/P21, and found a significant dose-dependent increase in the expression levels of both P53 and P21 proteins *in vitro* and *in vivo*. In addition, the P21 protein plays a role as an inhibitor of cell cycle progression, as P21 binds to the cell cycle protein kinase complex and inhibits its kinase activity, which is also closely related to the cycle regulation of icaritin (Xiong et al., 1993).

Senescence is mainly associated with G0/G1 arrest, however, our experimental results showed that icaritin-induced nasopharyngeal carcinoma cell lines were blocked in S phase and positive for SA-β-Gal staining, which is a very interesting phenomenon and warrants further exploration of the mechanism of icaritin-induced senescence. Studies have shown that senescence can be triggered by multiple stimuli, such as oxygen reactive substances and DNA damage (Ma et al., 2019; Sailor et al., 2022; Yang et al., 2022). We speculate that icaritin-induced senescence may be related to the above factors, and the increased expression of the DNA damage marker protein γ-H2AX further supports our speculation. ROS is closely associated with DNA damage, cell senescence, and other cellular damages (Maciel-Baron et al., 2018; Xiao et al., 2022). Several studies have shown that icaritin can promote ROS production in tumor cells, and our flow cytometry results showing that icaritin can enhance ROS levels in nasopharyngeal carcinoma cells are consistent with the findings of these studies (Li et al., 2016; Chen et al., 2019). Inhibition of ROS enhanced cell viability and reduced the proportion of senescent cells, suggesting that icaritin exerts anti-tumor effects through ROS release.

## Conclusion

Icaritin has been shown to have effective anti-tumor properties in both *in vitro* and *in vivo* investigations. Combining with network pharmacological analysis and cellular validation, we found that icaritin exerts anti-tumor effects by promoting ROS production, damaging DNA, and inducing S-phase arrest to inhibit proliferation of nasopharyngeal carcinoma cells. The results provide a new direction for exploring the mechanism of icaritin and its use in treatment of nasopharyngeal carcinoma.

## Data Availability

The original contributions presented in the study are included in the article/Supplementary Material, further inquiries can be directed to the corresponding authors.
