# Corrigendum: Exploring the pharmacological mechanisms of icaritin against nasopharyngeal carcinoma *via* network pharmacology and experimental validation

**DOI:** 10.3389/fphar.2023.1140018

**Published:** 2023-01-31

**Authors:** Minglu Liu, Tong Hu, Wenfeng Gou, Huajie Chang, Yanli Li, Yiliang Li, Daiying Zuo, Wenbin Hou, Shunchang Jiao

**Affiliations:** ^1^ Department of Medical Oncology, The First Medical Centre, Chinese People’s Liberation Army General Hospital, Beijing, China; ^2^ Tianjin Key Laboratory of Radiation Medicine and Molecular Nuclear Medicine, Institute of Radiation Medicine, Peking Union Medical College and Chinese Academy of Medical Sciences, Tianjin, China; ^3^ Department of Pharmacology, Shenyang Pharmaceutical University, Shenyang, China

**Keywords:** icaritin, nasopharyngeal carcinoma, ROS, senescence, network pharmacology

In the published article, there was an error in the **Author list**. The corrected author list appears below:

“Minglu Liu^1^, Tong Hu^2,3^, Wenfeng Gou^2^, Huajie Chang^2^, Yanli Li^2^, Yiliang Li^2^, Daiying Zuo^3^, Wenbin Hou^2,^*, Shunchang Jiao^1,^*”

The authors apologize for this error and state that this does not change the scientific conclusions of the article in any way. The original article has been updated.

