# Supplementary material for: Exploring the pharmacological mechanisms of icaritin against nasopharyngeal carcinoma via network pharmacology and experimental validation
Source: Front Pharmacol. 2022 Nov 18;13:993022. doi: 10.3389/fphar.2022.993022 (PMC9715612; doi:10.3389/fphar.2022.993022)
Supplement: Supplementary file 1 [file DataSheet1.PDF]

Supplementary Material

1. Supplementary Figures

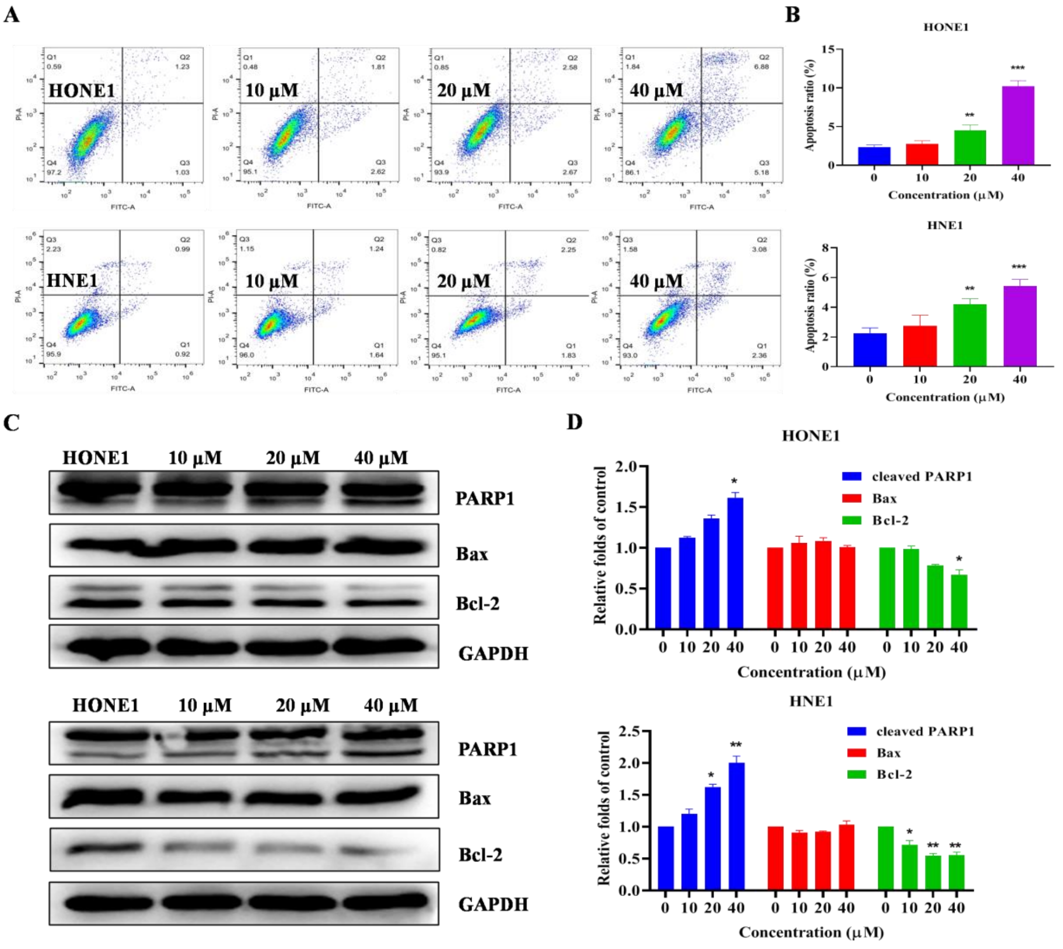

SUPPLEMENTAL FIGURE 1 | Icaritin induces slight apoptosis in HONE1 and HNE1 cells. **(A)** Apoptosis of HONE1 and HNE1 cells after treatment with different concentrations of icaritin for 48 h and measured using flow cytometry with Annexin V-FITC staining. **(B)** The percentage of apoptosis in HONE1 and HNE1 cells. **(C)** Changes in the expression of cell apoptosis-related proteins in HONE1 and HNE1 cells treated with different concentrations of icaritin for 48 h. **(D)** The quantitative data of western blot analysis. $^{*}p < 0.05$ versus control, $^{**}p < 0.01$ versus control, $^{***}p < 0.001$ versus control.

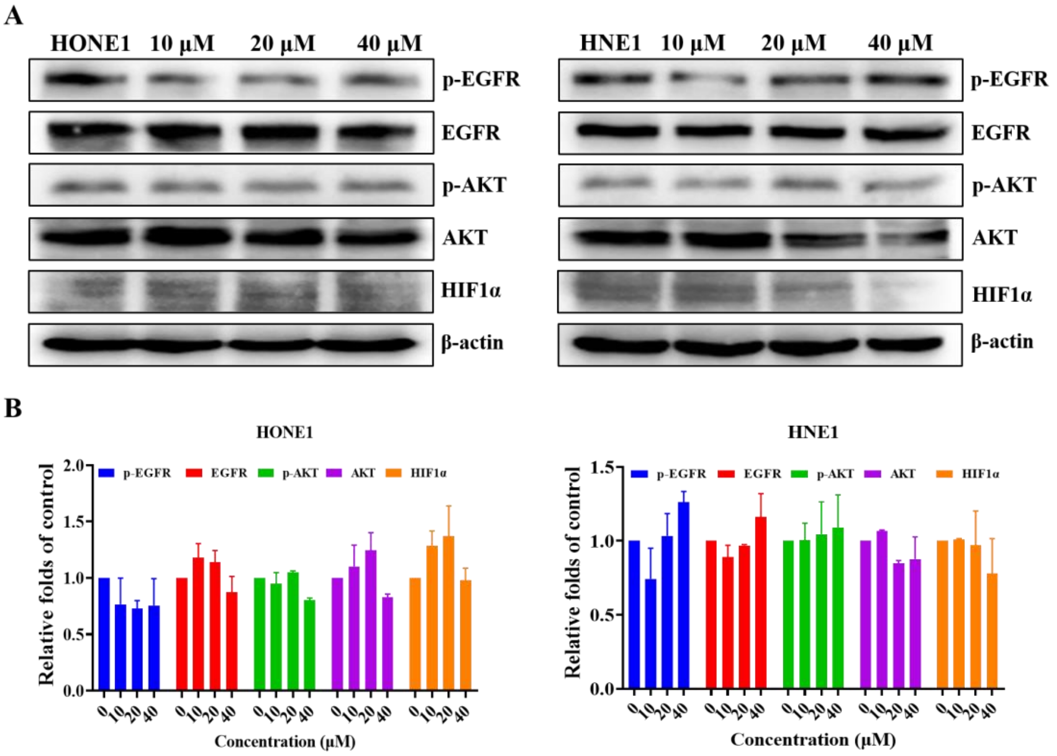

SUPPLEMENTAL FIGURE 2 | Icaritin has no significant effect on EGFR, PI3K/AKT pathway proteins. **(A)** The expression of EGFR, PI3K/AKT pathway proteins in HONE1 and HNE1 cells treated with icaritin (10, 20, or 40 μM) for 48 h was analyzed by western blot. **(B)** The quantitative data of Western blot analysis. $^{*}p < 0.05$ versus control, $^{**}p < 0.01$ versus control, $^{***}p < 0.001$ versus control.

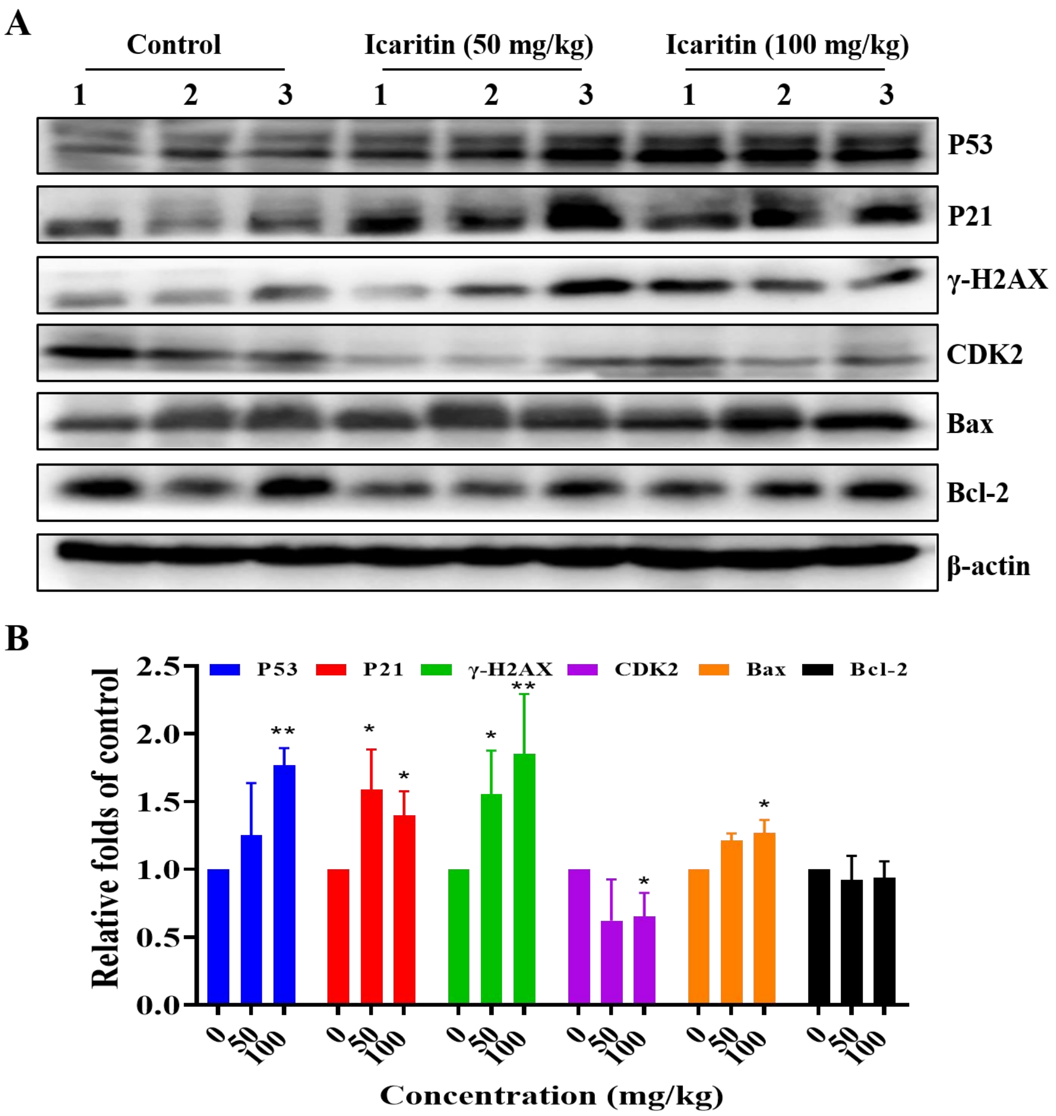

SUPPLEMENTAL FIGURE 3 | Icaritin enhances the expression of senescence-associated proteins in tumor tissues. **(A)** The expression of the senescence-related protein in HONE1 tumor tissues treated with icaritin (50 or 100 mg/kg) was analyzed by western blot. **(B)** The quantitative data of Western blot analysis. $^{*}p < 0.05$ versus control, $^{**}p < 0.01$ versus control versus control.

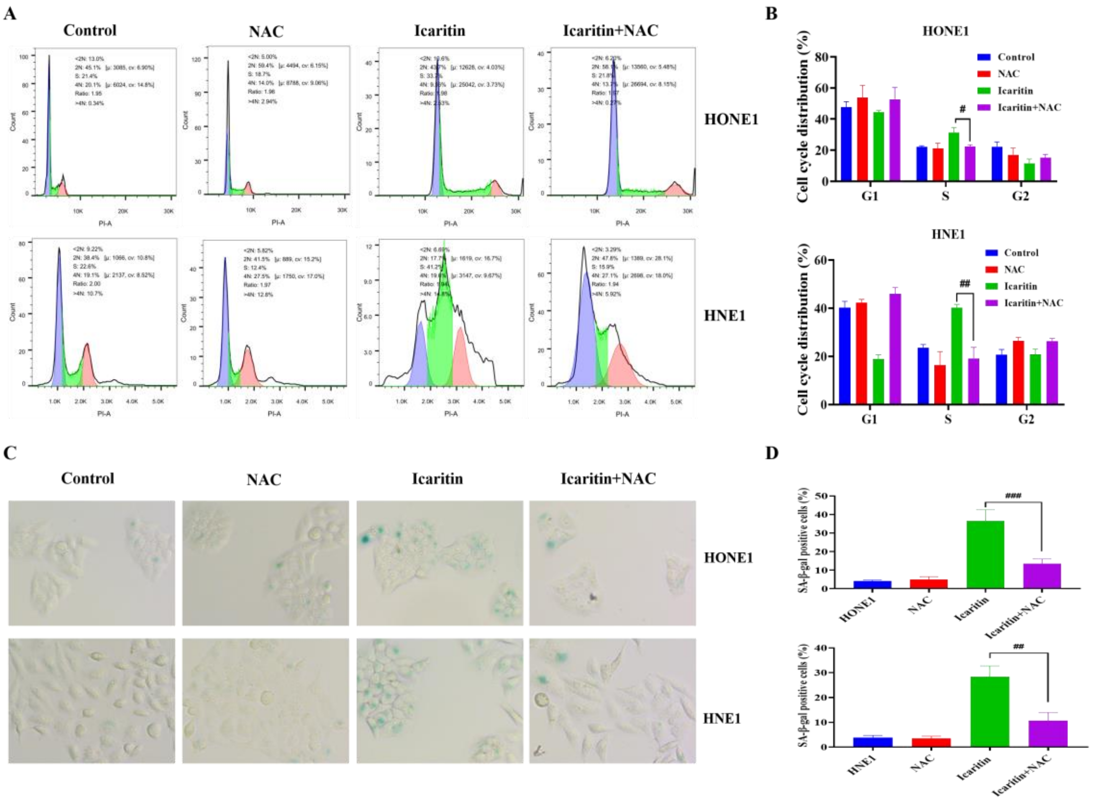

SUPPLEMENTAL FIGURE 4 | NAC reversed the icaritin-induced S-phase arrest and senescence in HONE1 and HNE1 cells. **(A)** The cell cycle distribution of HONE1 and HNE1 cells after treatment with icaritin (20 μM), NAC (5 μM), or both for 24 h and measured by flow cytometry with PI staining. **(B)** The percentage of cells in specific cell cycle phases. **(C)** Representative photomicrographs of SA-β-Gal staining in HONE1 and HNE1 cells after treatment with icaritin (20 μM), NAC (5 μM), or both for 24 h. **(D)** The percentage of SA-β-Gal positive cells. $^{\#}p < 0.05$, $^{\#\#}p < 0.01$, $^{\#\#\#}p < 0.001$ versus icaritin.

2. Supplementary Table

Supplementary Table 1. Common target genes of icaritin and nasopharyngeal carcinoma

NO	Uniport ID	Gene	Description
1	P24385	CCND1	G1/S-specific cyclin-D1
2	P04626	ERBB2	Receptor tyrosine-protein kinase erbB-2
3	P35222	CTNB1	Catenin beta-1
4	P00533	EGFR	Epidermal growth factor receptor
5	P31749	AKT1	RAC-alpha serine/threonine-protein kinase
6	Q16611	BAK	Bcl-2 homologous antagonist/killer
7	P10415	BCL2	Apoptosis regulator Bcl-2
8	P14679	TYRO	Tyrosinase
9	P53355	DAPK1	Death-associated protein kinase 1
10	P49841	GSK3B	Glycogen synthase kinase-3 beta
11	Q9UNQ0	ABCG2	Broad substrate specificity ATP-binding cassette transporter ABCG2
12	P07900	HS90A	Heat shock protein HSP 90-alpha
13	P08254	MMP3	Stromelysin-1
14	P35228	NOS2	Nitric oxide synthase, inducible
15	O15530	PDPK1	3-phosphoinositide-dependent protein kinase 1
16	P53350	PLK1	Serine/threonine-protein kinase PLK1
17	Q16790	CAH9	Carbonic anhydrase 9
18	P00374	DYR	Dihydrofolate reductase
19	P03372	ESR1	Estrogen receptor
20	P27695	APEX1	DNA-(apurinic or apyrimidinic site) endonuclease
21	Q07820	MCL1	Induced myeloid leukemia cell differentiation protein Mcl-1
22	P33527	MRP1	Multidrug resistance-associated protein 1
23	P27986	P85A	Phosphatidylinositol 3-kinase regulatory subunit alpha
24	P43405	KSYK	Tyrosine-protein kinase SYK
25	P06493	CDK1	Cyclin-dependent kinase 1
26	Q00535	CDK5	Cyclin-dependent-like kinase 5

27	P13569	CFTR	Cystic fibrosis transmembrane conductance regulator
28	Q09472	EP300	Histone acetyltransferase p300
29	P56817	BACE1	Beta-secretase 1
30	Q8N1Q1	CAH13	Carbonic anhydrase 13
31	P10636	TAU	Microtubule-associated protein tau
32	Q15746	MYLK	Myosin light chain kinase, smooth muscle
33	P51955	NEK2	Serine/threonine-protein kinase Nek2
34	Q9NPH5	NOX4	NADPH oxidase 4
35	Q16875	F263	6-phosphofructo-2-kinase/fructose-2,6-bisphosphatase 3
36	P08183	MDR1	ATP-dependent translocase ABCB1
37	P11309	PIM1	Serine/threonine-protein kinase pim-1
38	P04054	PA21B	Phospholipase A2
39	P25105	PTAFR	Platelet-activating factor receptor
40	P11388	TOP2A	DNA topoisomerase 2-alpha
41	P00918	CAH2	Carbonic anhydrase 2
42	P06881	CALCA	Calcitonin gene-related peptide 1
43	O96017	CHK2	Serine/threonine-protein kinase Chk2
44	Q9UM73	ALK	ALK tyrosine kinase receptor
45	P37231	PPARG	Peroxisome proliferator-activated receptor gamma
46	P06401	PRGR	Progesterone receptor
47	P11511	CP19A	Aromatase
48	P00749	UROK	Urokinase-type plasminogen activator
49	P24941	CDK2	Cyclin-dependent kinase 2
50	Q00534	CDK6	Cyclin-dependent kinase 6
51	Q16678	CP1B1	Cytochrome P450 1B1
52	P68400	CSK21	Casein kinase II subunit alpha
53	P14625	ENPL	Endoplasmin
54	P05164	PERM	Myeloperoxidase
55	P09917	LOX5	Polyunsaturated fatty acid 5-lipoxygenase
56	P21964	COMT	Catechol O-methyltransferase

57	P09467	F16P1	Fructose-1,6-bisphosphatase 1
58	Q9GZT9	EGLN1	Egl nine homolog 1
59	Q04828	AK1C1	Aldo-keto reductase family 1 member C1
60	P08238	HS90B	Heat shock protein HSP 90-beta
61	Q9NZJ5	E2AK3	Eukaryotic translation initiation factor 2-alpha kinase 3
62	O76074	PDE5A	cGMP-specific 3',5'-cyclic phosphodiesterase
63	P21397	AOFA	Amine oxidase [flavin-containing] A
